# Spatiotemporal Patterns of Substance P-Bound MRGPRX2 Reveal a Novel Connection Between Macropinosome Resolution and Secretory Granule Regeneration in Mast Cells

**DOI:** 10.3389/fimmu.2022.892239

**Published:** 2022-06-28

**Authors:** Pia Lazki-Hagenbach, Elisabeth Kleeblatt, Hydar Ali, Ronit Sagi-Eisenberg

**Affiliations:** ^1^Department of Cell and Developmental Biology, Sackler Faculty of Medicine, Tel Aviv University, Tel Aviv, Israel; ^2^Department of Basic and Translational Sciences, School of Dental Medicine, University of Pennsylvania, Philadelphia, PA, United States; ^3^Sagol School of Neuroscience, Tel Aviv University, Tel Aviv, Israel

**Keywords:** mast cell, MRGPRX2, substance P, endocytosis, autophagy, macropinocytosis, secretory granules

## Abstract

MRGPRX2, the human member of the MAS-related G protein coupled receptors (Mrgprs), serves as the cellular target of human mast cells (MCs) for innate ligands, including neuropeptides and antimicrobial peptides. In addition, MRGPRX2 also functions as the receptor for multiple FDA-approved drugs. As such, MRGPRX2 is a mediator of MC responses in neurogenic inflammation, host defense and pseudoallergy. We analyzed the spatiotemporal patterns of MRGPRX2 following its binding of the neuropeptide substance P (SP). Herein, we show that MRGPRX2 internalizes *via* both endocytosis and macropinocytosis, followed by its distribution between a perinuclear region and the secretory granules (SGs). Further, we show that MRGPRX2-containing macropinosomes undergo resolution by a mechanism that involves dynamin and LC3, giving rise to the incorporation of both LC3 and MRGPRX2 into the SGs. SP then promotes the acidification of the LC3-associated SGs, presumably by stimulating their fusion with lysosomes. Taken together, our results reveal a unique mode of MRGPRX2 trafficking that complements endocytosis and involves macropinocytosis, autophagic machinery-assisted macropinosome resolution and receptor delivery to the SGs.

## Introduction

While best known for their involvement in immunoglobulin E (IgE)-triggered allergy (1), mast cells (MCs) are key players in innate immune responses (2–4). Indeed, their strategic location in tissues that interface the external environment, jointly with their IgE-independent responses to multiple stressors, have marked them as sentinel cells and first responders to external insults (5). Both IgE-dependent and independent immune responses of the MCs are caused by the release of inflammatory mediators, part of which are pre-stored in cytoplasmic secretory granules (SGs) and released during regulated exocytosis (6, 7). Others, including lipid mediators, cytokines and chemokines are released following their *de novo* synthesis (8). Taken together, these mediators accomplish the important roles played by MCs in health and disease.

In particular intriguing are the responses of a subset of MCs to a variety of ligands, including neuropeptides, antimicrobial peptides and toxins, that share commonly only their positive charge (9, 10). MC responses to this class of stimuli play major roles in host defense and neurogenic inflammation (11–13). Furthermore, included in this class of ligands are multiple FDA-approved drugs, that by triggering MC degranulation cause pseudo-allergic reactions (14). The underlying mechanism(s) of MC activation by these positively charged stimuli has remained enigmatic until the recent identification of the Mas-related G protein coupled receptors (Mrgprs) as their cellular targets (15). This recognition has placed Mrgprs, and in particular MRGPRX2, the human member of this family, at a central position in MC biology.

The cellular functions of G protein coupled receptors (GPCRs) are tightly linked with their cellular positioning. Internalization and post-endocytic trafficking may terminate GPCR signaling or may prolong or diverse it (16–18). Therefore, we have undertaken to decipher the spatiotemporal patterns of MRGPRX2, following its binding of the neuropeptide substance P, that by binding to MRGPRX2 mediates the neuro-immune responses of MCs, such as in itch and pain (12, 19). Here we report on the unique patterns of MRGPRX2 internalization, that involve macropinocytosis and LC3-assisted delivery to the SGs.

## Material and Methods

### Antibodies and Reagents

Anti-human MRGPRX2 antibody (cat no. 359002) and anti-HA.11 Epitope Tag antibody (cat no. 901513) were from Biolegend (San Diego, CA). Anti-syntaxin 3 antibody (cat no. ab133750), secondary Alexa Fluor^®^ 488 Goat Anti-Rabbit IgG H&L (cat no. ab150077, dilution 1:500) and secondary Alexa Fluor^®^ 647 Goat Anti-Mouse IgG H&L (cat no. ab150115) were from Abcam (Cambridge, UK). Substance P (cat no. S6883), pitstop2 (cat no. SML1169) and saponin (cat no. S4521-25G) were purchased from Sigma-Aldrich (St Louis, MO, USA). Cytochalasin D (cat no. 1233), dynasore (cat no. 2897) and EIPA (cat no. 3378) were from Tocris Bioscience (Minneapolis, MN, USA). Tetramethylrhodamine (TRITC)- labeled 70 kDa dextran was purchased from Thermo Fischer Scientific (cat no. D1818, Waltham, MA, USA).

### Plasmids Used in this Study

The following expression plasmids were used in this study: Neuropeptide Y (NPY)–mRFP and NPY Venus were a gift from Dr. U. Ashery (Tel Aviv University, Tel Aviv, Israel), NPY-CFP was a gift from Dr. P. Blinder, (Tel Aviv University, Tel Aviv, Israel). pEGFP-LC3 was a gift from Dr. A. Ashkenazi (Tel Aviv University, Tel Aviv, Israel), originally a gift from Tamotsu Yoshimori (Addgene plasmid # 21073; http://n2t.net/addgene:21073; RRID : Addgene_21073) and LC3-EGFP-mRFP (ptfLC3) was a gift from Dr. R. Pinkas Kramarski (Tel Aviv University, Tel Aviv, Israel), originally a gift from Dr. Tamotsu Yoshimori (Addgene plasmid # 21074; http://n2t.net/addgene:21074; RRID : Addgene_21074). Lifeact-GFP was a gift from Dr. B.-Z. Shilo (Weizmann Institute of Science, Rehovot, Israel) and pcDNA3-EGFP-Rac1-T17N was a gift from Dr. Gary Bokoch (Addgene plasmid # 12982; http://n2t.net/addgene:12982; RRID : Addgene_12982). pcDNA3-EGFP was a gift from Dr. J. Silvio Gutkind (University of California, San Diego, USA).

### Cell Culture

RBL cells stably expressing N-terminally tagged hemagglutinin (HA) human MRGPRX2 (RBL-MRGPRX2) were generated as previously described (20). Cells were maintained at 37°C in a humidified incubator with 5% CO_2_ in adherent cell cultures in low glucose DMEM (cat no. 01-050-1A, Biological Industries, Beit-Haemek, Israel) supplemented with 10% FBS (cat no. 12657, GIBCO, Grand Island, NY), 2 mM L-Glutamine (cat no. 03-020-1A, Biological Industries), 100 μg/mL streptomycin and 100 U/mL penicillin, 12.5 U/mL nystatin (Biological Industries) and 1 mg/mL of G418 (cat no. A1720, Sigma Aldrich). LAD-2 cells (a kind gift from Dr. A.S. Kirshenbaum and Dr. D. Metcalfe, Laboratory of Allergic Diseases, National Institute of Allergy and Infectious Diseases, National Institutes of Health, Bethesda, MD) were cultured in StemPro-34 (cat no. 10640-019, GIBCO) supplemented with 1x StemPro-34 Nutrient, 2 mM L-Glutamine (cat no. 03-020-1A, Biological Industries), 100 U/mL penicillin and 100 μg/mL streptomycin (cat no. 03-032-1B, Biological Industries), and 100 ng/mL hSCF (cat no. 300-07, Peprotech, Rocky Hill, NJ).

### Cell Transfection

Transient transfection of RBL-MRGPRX2 cells was performed as previously described (21). Briefly, RBL-MRGPRX2 cells (1.5 x 10^7^) were transfected with 30-50 µg cDNA by electroporation at 300 V for 20 ms, using an ECM 830 electroporator (BTX, Holliston, Mass, USA). The cells were immediately replated in either 24-well (1 x 10^5^ cells/well) tissue culture dishes for confocal imaging or in 6-well (0.2 x 10^6^ cells/well) tissue culture dishes for flow cytometry experiments containing growth medium and used within 20-24 h after transfection.

### Dextran Uptake Assay

RBL-MRGPRX2 cells (1 x 10^5^ cells/well) were incubated with 0.1 mg/mL of TRITC- labeled 70 kDa dextran (D1818, Thermo Fischer Scientific) in Tyrode’s buffer (10 mM HEPES [pH 7.4], 130 mM NaCl, 5 mM KCl, 1.4 mM CaCl_2_, 1 mM MgCl_2_, 5.6 mM glucose, and 0.1% BSA) for the desired time periods, without or with 10 µM SP, in 24-well dishes at 37°C and 5% CO_2_. Where indicated, cells were preincubated for 30 minutes (min), prior to SP trigger, with vehicle or with the indicated inhibitor(s). Cells were washed three times with ice-cold PBS and fixed for 20 min at room temperature (RT) with 4% paraformaldehyde (catalog no. 15710; Electron Microscopy Sciences, Hatfield, Pa) followed by permeabilization for 15 min with 0.1% Triton X-100, 5% FBS, and 2% BSA diluted in PBS. Subsequently, cells were labeled with mouse anti-HA (1:100 dilution) antibody for 1 hour (h) at RT, followed by three washes and a 1-h incubation with Alexa Fluor^®^ 647 Goat Anti-Mouse IgG H&L secondary antibody (1:500 dilution).

### Flow Cytometry Analysis

RBL-MRGPRX2 cells were washed three times in Tyrode’s buffer to remove dead, non-adherent cells. RBL-MRGPRX2 (5 x 10^5^) or LAD-2 (6 x 10^5^) cells were stimulated at 37°C and 5% CO_2_, in Tyrode’s buffer for the desired time periods. Cells were then washed three times with ice-cold PBS containing 0.5% BSA and 2 mM sodium azide (FACS buffer) followed by fixation in 4% paraformaldehyde for 15 min at RT. For analyses of cell surface MRGPRX2, cells were stained for 30 min at RT with anti-human MRGPRX2 antibody (1:300 dilution). Cells were subsequently washed three times and stained with Alexa Fluor^®^ 647-conjugated Goat Anti-Mouse IgG H&L secondary antibody (1:2000 dilution) for 30 min at RT, in the dark. For total receptor expression, cells were washed and fixed for 15 min at RT with 4% paraformaldehyde. Cells were subsequently washed and permeabilized with 0.1% saponin and 10% FBS containing PBS for 15 min at RT, after which the cells were washed and stored in the permeabilization buffer overnight at 4°C. The permeabilized cells were then stained for 30 min at RT with anti-human MRGPRX2 antibody (1:300 dilution), washed three times with the permeabilization buffer and stained with the secondary antibody as above. Cells were washed three times with FACS buffer and analyzed by flow cytometry using a CytoFLEX LX flow cytometer (Beckman Coulter, Indianapolis, IN). A minimum of 10,000 cells was acquired per sample. Cells were gated on single cells and were additionally gated on GFP-positive cells in experiments involving transient transfection with either dominant negative (DN) Rac1-GFP or control GFP. Data was analyzed using the FlowJo™ Software Version 10 (Treestar, Ashland, OR).

### Immunostaining and Laser Confocal Microscopy Analysis

RBL-MRGPRX2 (1 x 10^5^) and LAD-2 (0.6 x 10^5^) cells grown on untreated (RBL-MRGPRX2) or fibronectin (cat no. F1141, Sigma Aldrich, St Louis, MO, USA) coated (LAD-2) 12-mm round glass coverslips (thick #1; Thermo Scientific, Menzel-Gläser, Saarbrücken, Germany) were washed three times in Tyrode’s buffer, the cells were stimulated as indicated in the same buffer at 37°C and 5% CO_2_. The cells were subsequently washed three times with ice-cold PBS and fixed for 20 min at RT with 4% paraformaldehyde (cat no. 15710; Electron Microscopy Sciences, Hatfield, PA) in PBS. Fixed cells were permeabilized for 15 min at RT with 0.1% Triton X-100, 5% FBS, and 2% BSA diluted in PBS. Cells were then incubated for 1 h at RT with the desired primary antibodies followed by three washes and a 1-h incubation with the appropriate secondary antibody (1:500 dilution). After washing, cells were mounted in mounting medium (cat no. E18-18; Golden Bridge Life Science, Mukilteo City, WA) and analyzed with a Leica SP5 or SP8 TCS laser scanning confocal microscope (Leica, Wetzlar, Germany) equipped with a HyD detector and a ×63 oil/1.4 NA objective or a Zeiss LSM 710 confocal microscope equipped with a 63 x1.4 oil Plan-Apochromat objective. Colocalization analysis of NPY-CFP with immunostained MRGPRX2 was quantified as the Manders’ correlation coefficient with Costes’ automatic threshold using the JaCoP plugin of the extended ImageJ version Fiji (22–24). Three-dimensional reconstructions of z stacks (slice thickness ≤ 0.5µm for both fluorescent channels with ~ 10 slices) were performed by using Imaris software (Bitplane, Zurich, Switzerland). Macropinosomes were defined as circular, phase-bright organelles that dissociated from circular ruffles or peripheral ruffles in cells stimulated with SP. To determine the mean fluorescence intensity (MFI) of macropinosomes, the borderline of each macropinosome was outlined with the segmented line tool, saved as a region of interest (ROI) and the MFI was measured using the extended ImageJ version Fiji. The background MFI was subtracted from the MFI of the macropinosomes for each cell, and the MFIs for at least 20 cells/condition were averaged. Next, the MFI values of four randomly selected regions of interest at the periphery of cells triggered with SP for 30 min were selected, measured, and averaged. This averaged MFI values were used for normalization. The size of macropinosomes was calculated by drawing the borderline of each macropinosome with the segmented line tool, again saved as ROIs. The length of the ROIs was then measured using the extended ImageJ version Fiji (23). To determine the vesicles/cell images were converted into threshold images, the required channels were merged (using the AND or XOR operations of the image calculator) to compare the signal patterns and then analyzed with the ImageJ “analyze particle” macro (23).

### Statistical Analysis

Data were analyzed using GraphPad Prism Version 8.3.0 for Windows (GraphPad Software, La Jolla, CA). One-way analysis of variance (ANOVA) with repeated measures followed by Dunnett’s post-test or Student’s or Welch’s t-test was used for comparing means, respectively according to the statistic requirements. Results were considered significant when P values were smaller than 0.05.

## Results

### MRGPRX2 Internalizes *via* Two Distinct Pathways

**Figure 1:**
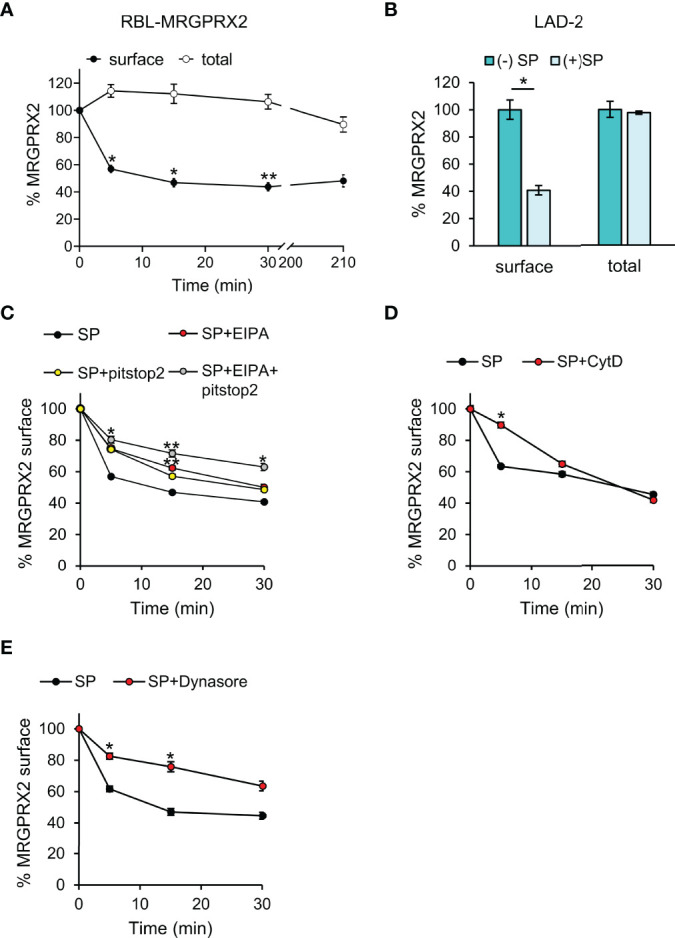
MRGPRX2 internalizes into non-recycling and non-degradative compartment(s). RBL-MRGPRX2 cells **(A, C–E)** or LAD-2 cells **(B)** were incubated for 30 min with either vehicle **(A, B)**, or 20 µM pitstop2, 50 µM EIPA or their combination **(C)**, or 10 µM CytD **(D)**, or 80 µM dynasore **(E)**, as indicated. Cells were subsequently triggered with 10 µM SP for the indicated time periods **(A, C-E)** or for 30 min **(B)** and stained with anti-MRGPRX2 antibodies in the absence (**A**, closed circles, **B–E**) or presence of 0.1% saponin (**A**, open circles, **B**). Cells were analyzed by flow cytometry as described under Materials and Methods. Cell surface and total receptor expression are presented as the percentage of cell surface or total MRGPRX2 expression in the absence of trigger. Data are means ± SEM (n = 5-9). Statistical significance was determined by one–way ANOVA, followed by Dunnett’s post-test, for A - C. *P < 0.05, **P < 0.01, and *P*< 0.05 for 5 min SP vs. 5 min SP +pitstop2, *P*< 0.01 for 5 min SP vs. 5 min SP + EIPA and *P*< 0.05 for 15 min SP vs. 15 min SP + pitstop2. Statistical significance was determined by unpaired two-tailed Student’s t-test for B, D and E *P[surface (–):SP vs. (+)SP] = 0.0156, *P[5 min: SP vs. SP+CytD] = 0.019, *P[5 min: SP vs. SP + dynasore] = 0.013, *P[15 min: SP vs. SP + dynasore] = 0.019.

Given the profound differences between the human MRGPRX2 and its rodent orthologs, we used the RBL-MRGPRX2 cells (19), that stably express the human MRGPRX2, as our model. We have recently validated the authenticity of this model by demonstrating that the ectopically expressed MRGPRX2 in RBL cells retains the authentic signaling elicited by the endogenously expressed MRGPRX2 in the human LAD-2 cells (21). Furthermore, unlike naïve RBL cells, RBL-MRGPRX2 cells have acquired responsiveness to MRGPRX2 ligands, this model allows measuring the exclusive responses of MRGPRX2 to ligands, such as SP, that additionally bind to canonical receptors, such as Neurokinin 1 (NK1), that is endogenously expressed in some MCs. Consistent with our previous results (25), flow cytometric analyses (FACS) revealed a reduction in the cell surface expression of MRGPRX2 in response to SP trigger (**Figure 1A** and **Supplementary Figure 1**). However, no significant changes were noted in the total amount of receptor (**Figure 1A**), implying that half of the total amount of the receptor either remains at the plasma membrane or internalizes, but recycles back to the plasma membrane. However, half of the total amount of receptor, which internalizes in response to SP stimulation, is targeted to an internal, non-degradative compartment(s), and is retained intracellularly, without recycling back to the plasma membrane (**Figure 1A**). A similar distribution was also noted for the endogenously expressed MRGPRX2 in LAD-2 cells, in which 40% of the total amount of receptor localized to the cell surface following 30 min of cell stimulation with SP, while 60% of the receptor could only be immunostained after cell permeabilization, implying its retainment in an intracellular compartment (**Figure 1B**).

Further analysis of the first phase of MRGPRX2 internalization in RBL-MRGPRX2 cells revealed that MRGPRX2 endocytosis was sensitive to pitstop2, an inhibitor of both clathrin-dependent endocytosis and clathrin-independent and Arf6-dependent endocytosis (26, 27) (**Figure 1C**). Thus, following a 5-min trigger with SP, 44% of MRGPRX2 internalized in the absence of pitstop2, but only 26% in its presence (**Figure 1C**). However, with increasing time of trigger, pitstop2 became less efficient, whereby approximately 60% of MRGPRX2 internalized following 30 min of SP trigger in the absence of inhibitor and 52% in its presence (**Figure 1C**). MRGPRX2 internalization also displayed a similar pattern of sensitivity to cytochalasin D (CytD), an inhibitor of actin polymerization (28), significantly inhibiting MRGPRX2 internalization following a 5-min trigger with SP, but displaying no inhibition following 30 min SP trigger (**Figure 1D**). Therefore, collectively, these results suggested that another pathway, alternative to endocytosis, exists for MRGPRX2 internalization.

Cell surface expression of MRGPRX2 was also reduced by dynasore, an inhibitor of dynamin, that executes membrane fission events (29) (**Figure 1E**). Furthermore, consistent with the more general role of dynamin in controlling internalization pathways, the inhibitory effect of dynasore was evident also at longer periods of SP trigger (**Figure 1E**).

**Figure 2:**
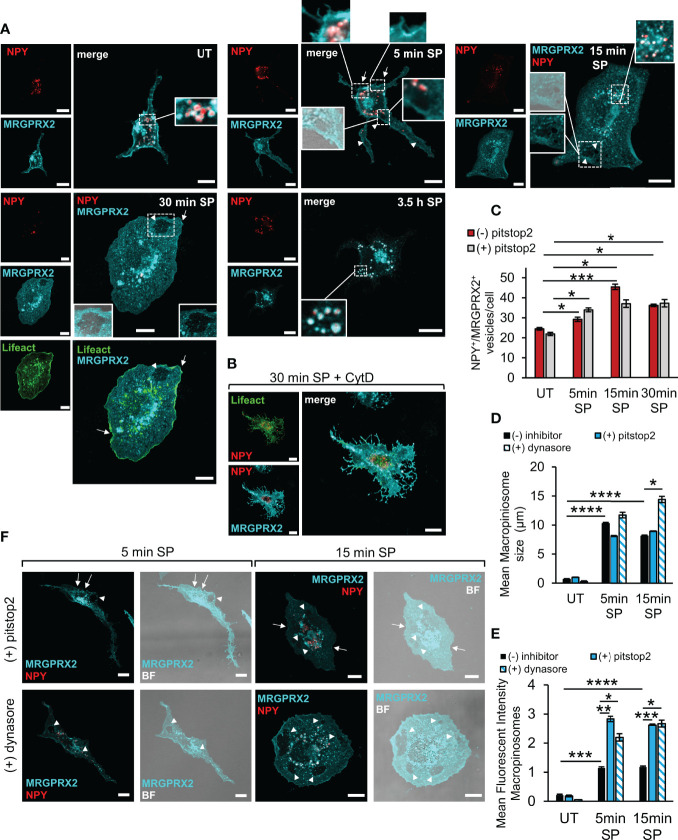
Modes of MRGPRX2 internalization. RBL-MRGPRX2 cells, transiently transfected with NPY-mRFP (red) or NPY-mRFP and Lifeact-GFP (green), as indicated, were either left untreated (UT), or triggered with 10 µM SP for the indicated time periods, in the absence of inhibitor **(A, C–E)**, or following preincubation for 30 min with 10 µM CytD **(B)**, 20 µM pitstop2 **(C, D–F)** or 80 µM dynasore **(D–F)**, as indicated. Cells were immunostained with anti-HA antibodies, followed by Alexa Fluor^®^ 647-conjugated secondary antibodies (pseudo colored cyan). Enlargements correspond to the boxed areas. Macropinosomes are shown by the overlap with the brightfield (BF) image. Arrows point to MRGPRX2 that is localized to cell ruffles. Arrowheads point to macropinosomes. Scale bars = 10µm. The mean number of vesicles/cell that are double positive for NPY-mRFP and MRGPRX2 (NPY^+^/MRGPRX2^+^) **(C)**, the mean size of macropinosomes **(D)**, and the Mean Fluorescent Intensity (MFI) of macropinosome-associated MRGPRX2 **(E)**, were calculated as described under Materials and Methods. Results are the means ± SEM from three independent experiments for dynasore and four independent experiments for pitstop2, each experiment including at least 20 cells/condition. Statistical significance was determined by one–way ANOVA, followed by Dunnett’s post-test for C *P < 0.05, **P < 0.01 and ***P < 0.001. Statistical significance was determined by unpaired two-tailed Student’s t-test for D, ****P[UT vs. 5 min SP] = 6.12552E-08, ****P[UT vs.15 min SP = 1.62931E-07, *P[15 min: SP vs. SP + dynasore] = 0.013, and for E, ***P[UT vs. 5 min SP] = 0.0001, ****P[UT vs. 15 min SP] = 2.01147E-05, *P[5 min: SP vs. SP + dynasore] = 0.042, **P[5 min: SP vs. SP + pitstop2] = 0.0017, *P[15 min: SP vs. SP + dynasore] = 0.0171, ***P[15 min: SP vs. SP + pitstop2] = 4.6873E-06).

In agreement with our previous results (21), close inspection of the receptor by high resolution confocal microscopy revealed that in resting cells, the receptor distributed between the plasma membrane and an internal fraction, part of which was perinuclear, reminiscent of the Golgi/TGN, and part resided at vesicles, which we have previously identified as the SGs, based on their labelling with the SG marker, NPY-mRFP (30) (**Figure 2A**). Confocal images of the cells also revealed marked alterations in cell morphology that occurred following their stimulation with SP (**Figure 2A**). The latter included cell flattening and formation of membrane ruffles and macropinosomes, that were identified by brightfield images and confirmed by their proximity to actin ruffles, visualized by the expression of Lifeact-GFP, an actin marker (**Figure 2A**). Furthermore, some of the plasma membrane-localized receptor concentrated at what appeared like sites of macropinosome formation from the membrane ruffles, and some localized to the macropinosomes (**Figure 2A**). Following a longer period of SP trigger (i.e. 3.5 h), the majority of MRGPRX2 distributed between the perinuclear region and the NPY-mRFP-labelled SGs (**Figure 2A**). Similar results were also observed in the LAD-2 cells, in which at the end of 30 min stimulation by SP, the internalized receptor was detected in the vicinity of macropinosomes and SGs, which were stained by anti-syntaxin 3 antibodies (**Supplementary Figure 2**). In contrast, MRGPRX2 localized to membrane protrusions, associated with actin ruffles, in RBL-MRGPRX2 cells that were triggered by SP for 30 min in the presence of CytD (**Figure 2B**). Quantitative analysis of the confocal images revealed significant increases in MRGPRX2 association with the SGs (**Figure 2C**), size of the cellular macropinosomes (**Figure 2D**) and the extent of MRGPRX2 association with macropinosomes (**Figure 2E**), in response to cell triggering with SP.

Imaging of cells that were triggered for 5 min with SP in the presence of pitstop2 demonstrated a significant increase in the amount of macropinosome-associated MRGPRX2 (**Figures 2E, F**), with no significant change in the SG-localized MRGPRX2 (**Figure 2C**). Therefore, these results implied that pitstop2-sensitive endocytosis and macropinocytosis may represent two interchangeable modes of MRGPRX2 internalization, whereby inhibition of one pathway, i.e. endocytosis by pitstop2, shifts the receptor to the alternative macropinocytic pathway.

We also analyzed the influence of dynasore on the distribution of MRGPRX2 and found that in a similar manner to the effect of pitstop2, also dynasore significantly increased the amount of macropinosome-associated MRGPRX2 (**Figures 2E, F****)**. Furthermore, dynasore significantly increased the mean size of the cellular macropinosomes as well (**Figures 2D, F**).

### SP Stimulates Macropinocytosis

**Figure 3:**
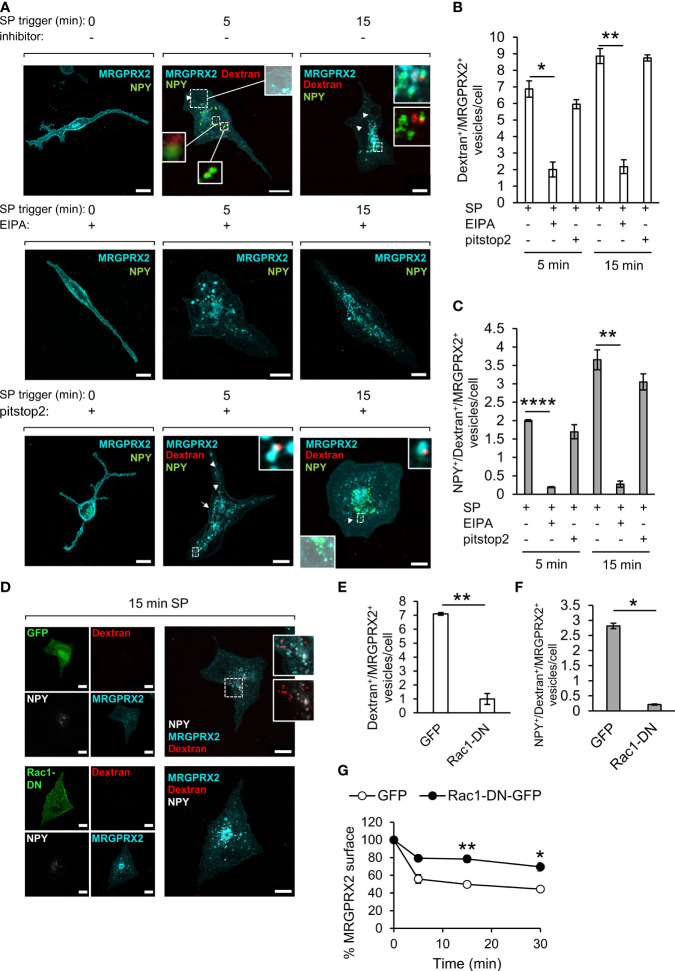
MRGPRX2 internalizes by macropinocytosis. RBL-MRGPRX2 cells, transiently transfected with NPY-Venus (green) **(A)**, or co-transfected with NPY-CFP (pseudo colored white) and either GFP or Rac1-DN-GFP (green) **(D)**, were triggered with 10 µM SP in the presence of 0.1 mg/ml TRITC-Dextran (red) for the indicated time periods, in the presence of vehicle **(A, D)** or following 30 min pre-incubation with either 50 µM EIPA or 20 µM pitstop2, as indicated **(A)**. Cells were immunostained with anti-HA antibodies followed by Alexa Fluor^®^ 647-conjugated secondary antibodies (pseudo colored cyan). Enlargements correspond to the boxed areas. Macropinosomes are shown as the overlap with the brightfield images. Arrows point to MRGPRX2 position at sites of macropinosome formation and arrowheads point to macropinosomes. Scale bars = 10µm. The mean number of vesicles/cell that are double positive for Dextran and MRGPRX2 (Dextran^+^/MRGPRX2^+^) **(B, E)**, or triple positive for NPY, Dextran and MRGPRX2 (NPY^+^/Dextran^+^/MRGPRX2) **(C, F)**, was calculated for cells that were triggered for the indicated time periods with SP (15 min for **E** and **F**), in the absence or presence of the indicated inhibitors or Rac1-DN. Results are means ± SEM from three **(D–F, G)** or four **(A–C)** independent experiments, with each experiment including 15-20 cells/condition. The effect of Rac1-DN expression on SP-induced internalization of MRGPRX2 was quantified by flow cytometry, by measuring the cell surface amount of MRGPRX2 gated on GFP-positive single cells. Results are presented as the percentage of the amount of cell surface MRGPRX2 in the absence of stimulation by SP **(G)**. Data are means ± SEM of four independent experiments. Statistical significance was determined by unpaired two-tailed Welch’s t-test. P values for Dextran^+^/MRGPRX2^+^ vesicles/cell: *P[5 min: SP vs. SP+ EIPA] = 0.0271, **P[15 min: SP vs. SP + EIPA] = 0.0026, **P[GFP vs. Rac1-DN-GFP] = 0.009. P values for NPY^+^/Dextran^+^/MRGPRX2^+^ vesicles/cell: **P[15 min: SP vs. SP + EIPA] = 0.01, ****P[5 min: SP vs. 5 min SP + EIPA] = 2.996x10^6, *P[GFP vs. Rac1-DN-GFP] = 0.018. P values for MRGPRX2 surface expression are **P[5 min: SP/GFP vs. SP/Rac1-DN-GFP] = 0.01, *P[15 min: SP/GFP vs. SP/Rac1-DN-GFP] = 0.0358.

To gain direct evidence for the involvement of macropinocytosis in MRGPRX2 internalization, we allowed the cells to take up TRITC-dextran (70 kDa), a commonly used probe for measuring macropinocytosis (31), and compared its location with the location of the internalized MRGPRX2. The results of these experiments revealed that SP stimulated the uptake of TRITC-dextran, which could be completely inhibited by EIPA, a Na^+^/H^+^ exchange inhibitor, that is known to inhibit macropinocytosis (32, 33) (**Figures 3A, B****)**. These results therefore demonstrated that SP can stimulate macropinocytosis. Furthermore, MRGPRX2 localized to the same dextran-containing vesicles (**Figures 3A, B**), part of which also contained NPY-Venus (**Figures 3A, C**), revealing a connection between MRGPRX2-positive macropinosomes and the SGs. In sharp contrast to the profound effect of EIPA, the uptake of TRITC-dextran was not affected by pitstop2 (**Figures 3A–C**), indicating that this alternative pathway of MRGPRX2 internalization was insensitive to this inhibitor, consistent with its ability to compensate for MRGPRX2 internalization by endocytosis (**Figure 2E**). Indeed, analysis of the cell surface expression of MRGPRX2 by flow cytometry revealed that EIPA was as potent as pitstop2 in inhibiting MRGPRX2 internalization (**Figure 1B**). Furthermore, combining EIPA with pitstop2 further inhibited internalization, supporting the notion of MRGPRX2 internalization *via* both pitstop2-sensitive endocytosis and EIPA-sensitive macropinocytosis (**Figure 1B**).

To substantiate the role of macropinocytosis in MRGPRX2 internalization even further, we also examined the impact of expression of a dominant negative (DN) mutant of Rac1 [i.e. Rac1(T17N)], a key player of actin rearrangements during macropinocytosis (34, 35), on the internalization of MRGPRX2, using the internalization of dextran as a positive control. Expectedly, DN Rac1 inhibited the uptake of TRITC-dextran (**Figure 3D**) and accordingly significantly reduced the number of internal vesicles that were double positive for dextran and MRGPRX2 (**Figure 3E**) or triple positive for dextran, MRGPRX2 and NPY-CFP (**Figure 3F**), which like NPY-mRFP and NPY-Venus, serves as a SG marker. Finally, DN Rac1 significantly retained the cell surface expression of MRGPRX2 (**Figure 3G**), thus demonstrating its capacity to inhibit SP-stimulated internalization of MRGPRX2.

### Trafficking of MRGPRX2 to the SGs Involves the Autophagic Machinery

**Figure 4:**
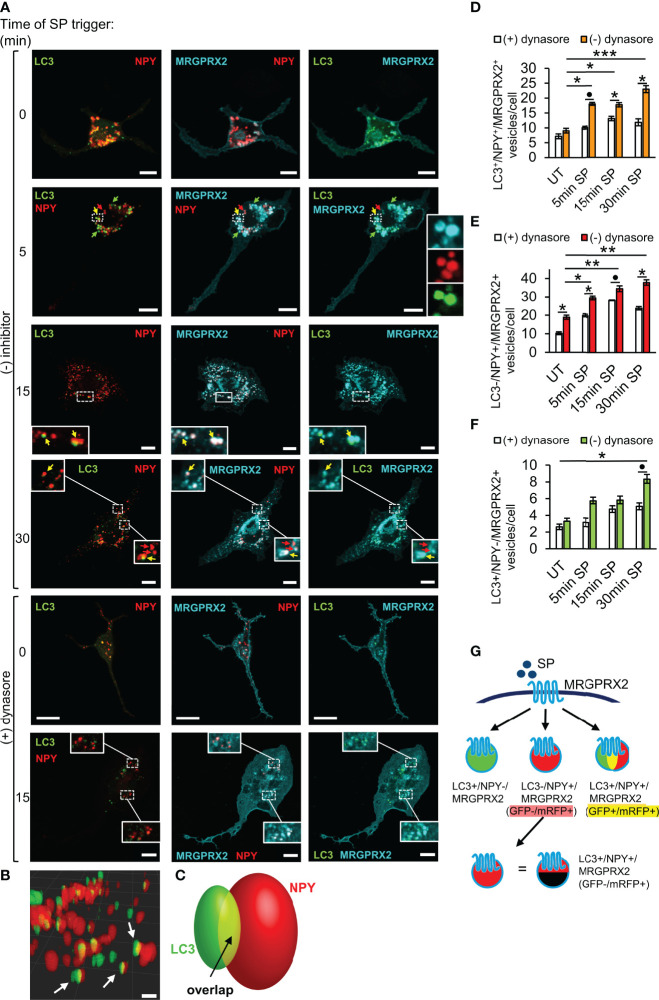
LC3 and dynamin assist MRGPRX2 trafficking to SGs. RBL-MRGPRX2 cells, transiently co-transfected with NPY-mRFP (red) and LC3-GFP (green), were triggered for the indicated time periods with 10 µM SP, in the presence of vehicle or following 30 min pre-incubation with 80 µM dynasore, as indicated **(A)**. Cells were immunostained with anti-HA antibodies followed by Alexa Fluor^®^ 647-conjugated secondary antibodies (pseudo colored cyan). Yellow arrows point to MRGPRX2 that is localized to NPY-mRFP and LC3-positive SGs, red arrows point to NPY-mRFP-positive, but LC3 negative SGs, and green arrows point to LC3 positive, but NPY-mRFP negative vesicles. Enlargements correspond to the boxed areas. Scale bars = 10µm. Confocal images of cells that were activated for 15 min with SP, were 3-dimensionally reconstructed by using Imaris software (scale bars 2 µm) **(B)**. Arrows point to LC3^+^/NPY^+^ vesicles. A scheme of a 3-dimensionally reconstructed LC3^+^/NPY^+^ vesicle is shown in **(C)**, showing that some of the fluorescence of LC3-GFP overlaps with NPY-mRFP (yellow). The average number of LC3^+^/NPY^+^/MRGPRX2^+^ **(D)**, LC3^-^/NPY^+^/MRGPRX2^+^ **(E)**, or LC3^+^/NPY^-^/MRGPRX2^+^ **(F)** vesicles/cell was calculated using the ImageJ software and is presented as the mean ± SEM from three to six independent experiments, with each experiment including at least 20 cells/condition. Statistical significance was determined by one-way ANOVA, followed by Dunnett’s post-test, for comparison of UT with different time points of SP trigger in D-F: *P < 0.05, **P < 0.01, ***P < 0.001, or unpaired two-tailed Welch’s t-test comparison SP ± dynasore for D, ^*^P[LC3^+^/NPY^+^/MRGPRX2^+^ - 5 min: SP vs. SP + dynasore] = 0.0652, for E, *P[LC3^+^/NPY^+^/MRGPRX2^+^ - 15 min: SP vs. SP + dynasore] = 0.0220, *P[LC3^+^/NPY^+^/MRGPRX2^+^ - 30 min: SP vs. SP + dynasore] = 0.0365, and for F, *P[LC3^-^/NPY^+^/MRGPRX2^+^ - UT: SP vs. SP + dynasore] = 0.0191, *P[LC3^-^/NPY^+^/MRGPRX2^+^ - 5 min: SP vs. SP + dynasore] = 0.0238, ^*^P[LC3^-^/NPY^+^/MRGPRX2^+^ - 15 min: SP vs. SP + dynasore] = 0.1049 and *P[LC3^-^/NPY^+^/MRGPRX2^+^ - 5 min: SP vs. SP + dynasore] = 0.0216. A schematic presentation of the types of vesicles into which MRGPRX2 internalizes in response to SP trigger is shown in **(G)**.

We have previously shown that early endosomes fuse with the SGs in MCs (36), thus providing a possible mechanism for MRGPRX2 targeting to the SGs. However, our results, showing that inhibition of endocytosis by pitstop2 does not prevent MRGPRX2 targeting to the SGs (**Figure 2C**), strongly suggested that MRGPRX2 can reach the SGs also directly from macropinosomes. Recently, it was demonstrated that cells respond to plasma membrane injury by forming large macropinosomes at the repair site (37). These structures eventually become positive for the autophagy-related LC3B protein, followed by their shrinkage and fusion with lysosomes (37). Given the lysosomal nature of the MC SGs, the previous reports that documented the presence of LC3 on MC SGs (38, 39), and our recent findings on inhibition of MRGPRX2-stimulated secretion by autophagy targeting drugs (21), we were motivated to examine if LC3 plays a role in MRGPRX2 delivery from macropinosomes to the SGs. To this end, we monitored the intracellular distribution of MRGPRX2 in response to SP trigger, in cells that were co-transfected with NPY-mRFP and LC3-GFP. Analysis of the confocal images revealed that the vesicular fraction of MRGPRX2, which increased with the time of SP trigger, indeed resided on NPY-mRFP labelled SGs, however a fraction of the NPY-positive SGs also labelled positive for LC3-GFP (**Figures 4A–E**). A smaller fraction of intracellular MRGPRX2 localized to LC3-GFP-positive, but NPY-mRFP-negative vesicles (**Figure 4F**), that were smaller in size and resided in close proximity to the SGs (**Figures 4A, B**). Consistent with the reports on bone marrow derived MCs (38), these vesicles, that may correspond to autophagosomes, were present also in resting cells (**Figure 4A**). Three-dimensional reconstructions of the confocal images corroborated the existence of three types of vesicles (**Figure 4B**, and **Supplementary Video 1**), and suggested that a fusion event between LC3-positive vesicles and LC3-negative SGs gives rise to the formation of the LC3-positve SGs (**Figure 4C**). This fusion event is fast and therefore restricting the number of LC3-positive and NPY-negative vesicles.

To further decipher the connection between macropinocytosis and MRGPRX2 trafficking to the SGs, we tested the impact of dynasore, that inhibits endocytosis, on the cellular distribution of MRGPRX2 in the LC3-GFP-expressing cells. Consistent with its profound impact on the macropinosomes’ size (**Figure 2D**), dynasore significantly inhibited the association of MRGPRX2 with LC3-posivtive as well as LC3-negative SGs (**Figures 4A, D–F**). These results therefore implicated dynamin in playing a role in the delivery of MRGPRX2 from macropinosomes to the SGs.

**Figure 5:**
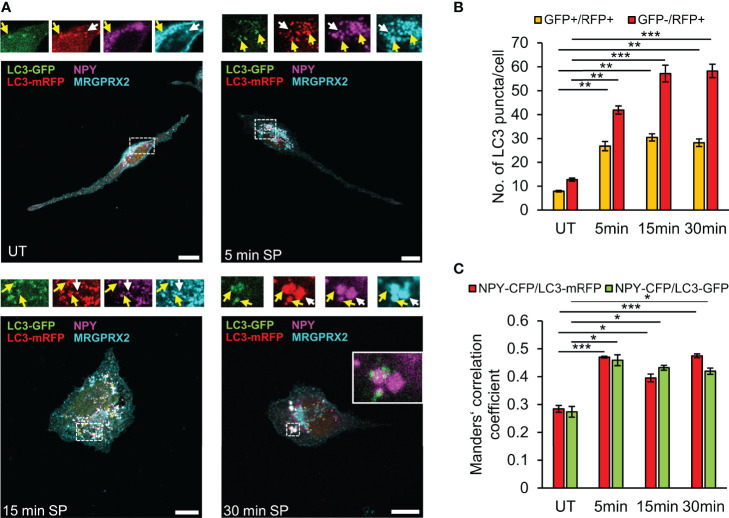
SP stimulates fusion of LC3-positive SGs with lysosomes. RBL-MRGPRX2 cells, transiently co-transfected with NPY-CFP (magenta) and LC3-GFP-mRFP (green and red), were either left untreated (UT) or triggered for the indicated time periods with 10 µM SP. Cells were subsequently immunostained with anti-HA antibodies followed by Alexa Fluor^®^ 647-conjugated secondary antibodies (pseudo colored cyan) **(A)**. Enlargements correspond to the boxed areas. Yellow arrows point to MRGPRX2 that is localized to LC3-positive-SGs (GFP^+^/mRFP^+^), and white arrows point to MRGPRX2 that is localized to LC3-autolysosome-SGs (GFP^-^/mRFP^+^). An example of the merge of NPY-CFP and LC3-GFP is shown in the inset of the 30 min image. Scale bars = 10µm. The average number of double positive GFP^+^/mRFP^+^ vesicles or single GFP^-^/mRFP^+^ vesicles was quantified using the ImageJ software and is presented as the mean ± SEM from five independent experiments, with each experiment including at least 20 cells/condition **(B)**. The extent of colocalization of NPY-CFP with LC3-mRFP or LC3-GFP was quantified (n = 3) and is presented as Mander’s correlation coefficient **(C)**. Statistical significance was determined by one-way ANOVA, followed by Dunnett’s post-test. *P < 0.05, **P < 0.01, *** *P*< 0.001.

GFP fluorescence is pH sensitive. Thus, while LC3-GFP is fluorescent in autophagosomes, this fluorescence quenches in autolysosomes that form following autophagosome fusion with lysosomes. The possibility therefore arises that the LC3-negative SGs (i.e. LC3^-^/NPY^+^) may still contain LC3-GFP, the fluorescence of which is quenched (**Figure 4G**). To address this possibility, we repeated our analyses of MRGPRX2 distribution in cells that were co-transfected with NPY-CFP and LC3-GFP-mRFP, a tandem fluorescent protein that is commonly used to monitor various stages of autophagy due to the pH insensitivity of mRFP. Thus, LC3-GFP-mRFP yields both green and red signals when located in the less acidic autophagosomes and only a red signal when located in the acidic autolysosomes (40). These studies demonstrated the presence of LC3-containing vesicles that were either double positive for GFP and mRFP, or only positive for mRFP (**Figure 5A**). Notably, the amount of both types of LC3-positive vesicles significantly increased in response to SP trigger (**Figure 5B**), indicating that they were formed by a dynamic and trigger-dependent process. Furthermore, with increasing time of SP trigger, the relative amount of GFP-negative/mRFP-positive vesicles increased, suggesting that SP stimulated their acidification, presumably by promoting their fusion with lysosomes (**Figure 5B**). NPY-CFP colocalized with both LC3-GFP-mRFP double positive and LC3-GFP-negative and mRFP-positive vesicles (**Figures 5A, C**), implying that both types of MRGPRX2-associated SGs contain LC3, but differ in their internal pH.

## Discussion

While internalization of GPCRs has been historically associated with signal termination, it is currently well established that GPCRs may continue signaling post their internalization. Moreover, their intracellular signaling patterns may differ from their plasma membrane signaling (18, 41). Therefore, deciphering the spatiotemporal regulation of GPCRs is central to understanding their functions. Given the important role played by MRGPRX2 in mediating MC innate immune and neurogenic responses, we have undertaken this study to investigate its route of trafficking in response to the binding of its neuropeptide ligand SP. We have previously shown that SP stimulates MRGPRX2 internalization (25), but the fate of the internalized receptor has not been characterized before. The first important finding that emerged from this study is the recognition that while a fraction of the internalized receptor may recycle back to the plasma membrane, approximately half of internalized MRGPRX2 is retained intracellularly, indicating its trafficking to a neither recycling nor degradative compartment. This pattern of intracellular targeting was not the result of overexpression, because we have previously shown that the level of MRGPRX2 expression in the stably transfected RBL-MRGPRX2 cells is even lower than its endogenous expression in LAD-2 cells (21). Furthermore, similar intracellular retention was also noted in the LAD-2 cells. Second, we demonstrate that MRGPRX2 internalizes *via* two parallel pathways, that can compensate each other, upon inhibition of one of them. Specifically, we show that alongside its internalization by an endocytic pathway, MRGPRX2 internalizes by macropinocytosis. This conclusion is supported by a number of observations, including the location of the receptor in macropinosomes, its colocalization with internalized dextran (70 kDa) and the inhibition of its targeting to macropinosomes by either EIPA or expression of DN Rac1, both known inhibitors of macropinocytosis (33, 35). The endocytic pathway of MRGPRX2 internalization is sensitive to pitstop2, an inhibitor of clathrin-dependent endocytosis and clathrin-independent and Arf6-dependent endocytosis (26, 27), thus implicating either one of these pitstop2-sensitive endocytic pathways in mediating MRGPRX2 internalization. However, the fact that MRGPRX2 internalization is only partially inhibited by the combination of pitstop2 and EIPA raises the possibility that an additional mode of clathrin-independent endocytosis that is resistant to pitstop2, might also be involved.

Macropinocytosis has been associated with innate immune cell surveillance and has been demonstrated to occur in macrophages and dendritic cells in response to microbe associated molecular patterns (42). This concept was recently extended by the finding that macropinocytosis complements endocytosis in internalizing the chemokine GPCR, CCR5 in macrophages (43). Our demonstration that MRGPRX2, a GPCR that mediates a broad spectrum of innate immune responses of MCs, also internalizes by both endocytosis and macropinocytosis strongly supports the recognition of macropinocytosis as a central mechanism in innate immunity. Whether macropinosomes comprise a site of intracellular signaling of MRGPRX2 is presently unknown and is the subject of our future research.

**Figure 6:**
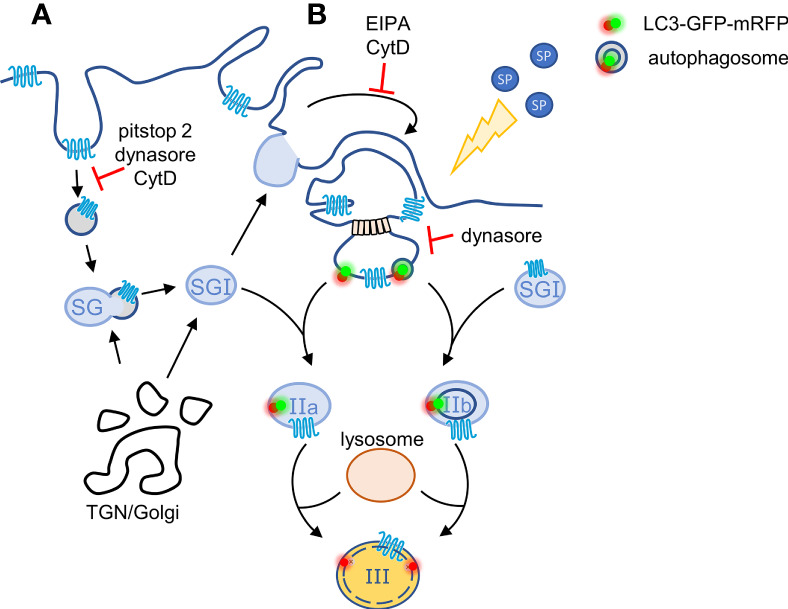
Model for the crosstalk between macropinosomes and the SGs. According to our model, plasma membrane-localized MRGPRX2 internalizes in response to cell stimulation by SP by two parallel mechanisms: **(A)** by endocytosis that is partially sensitive to pitstop2 and **(B)** by macropinocytosis. Endosomes may fuse with the SGs, thereby delivering MRGPRX2 to the SGs, whereas MRGPRX2-containing vesicles bud from macropinosomes by a mechanism that involves dynamin and either the binding of cytosolic or phagophore associated LC3, or fusion with autophagosomes. These vesicles then fuse with TGN-derived SGs (Type I) generating respectively, Type IIa or Type IIb, LC3-positive SGs. The latter then acidify, most likely by their fusion with lysosomes, by a SP-stimulated process, generating Type III SGs.

Analysis of the post endocytic trafficking of SP-bound MRGPRX2 revealed that the receptor distributes between a perinuclear region, reminiscent of the Golgi/TGN and the SGs. The latter intriguing location prompted us to investigate the route by which MRGPRX2 reaches the SGs. We have previously shown that MC SGs incorporate endocytic cargo by fusing with early endosomes (34). Thus, SG fusion with endosomes could provide a mechanism for MRGPRX2 targeting to the SGs. However, the finding that inhibition of endocytosis by pitstop2 does not prevent MRGPRX2 delivery to the SGs, suggested the existence of an alternative pathway that connects macropinocytosis, the second mechanism of MRGPRX2 internalization, with the SGs. Motivated to delineate such pathway, we hypothesized that macropinosome resolution may serve as a mechanism of regenerating SGs. We based our hypothesis on parallel mechanisms that exist during phagocytosis, where phagosome fragmentation leads to lysosome reformation (44), or repair of plasma membrane injury, during which LC3-mediated resolution of macropinosomes is linked with fusion with lysosomes (37). Indeed, our results reveal a novel link between macropinosomes, LC3 and the SGs. Specifically, we show that MRGPRX2 internalizes into LC3-positive vesicles that very rapidly acquire the patterns of LC3-GFP-positive-mRFP-positive SGs, followed by the patterns of LC3-GFP-negative-mRFP positive SGs. Furthermore, in the presence of dynasore, that blocks endocytosis, reduction in the amount of MRGPRX2 that is associated with these LC3-positive vesicles is accompanied by an increase in the macropinosome size. Therefore, taken together, our results are compatible with a model, whereby plasma membrane-localized MRGPRX2 internalizes in response to cell trigger by SP by both endocytosis, which may include both clathrin-dependent and clathrin-independent endocytosis, and by macropinocytosis, that may or may not be coupled to SG recapture (**Figure 6**). Endosomes then fuse with the SGs, thereby delivering MRGPRX2 to the SGs, whereas dynamin and LC3 promote the budding of macropinosome-derived vesicles leading to macropinosome resolution (**Figure 6**). These macropinosome-derived vesicles then fuse with NPY-mRFP labelled SGs (type I in our model, **Figure 6**), to form NPY positive and GFP-LC3-positive SGs (type II in the model, **Figure 6**), which then fuse with lysosomes to form NPY-positive/LC3-GFP-negative, but LC3-mRFP-positive, SGs (type III in the model, **Figure 6**). Therefore, this pathway provides not only a mode for incorporation of extracellular or membranal cargo into the SGs, but it may also serve as mechanism of regeneration of SGs by capturing part of the released cargo by macropinocytosis and incorporating it back into the SGs. Alternatively, macropinocytosis might be directly involved in the recapturing of SGs that have partially emptied their content during exocytosis. Macropinosomes may thereby facilitate the internalization of MRGPRX2, and its SG targeting by their subsequent fusion with non-degranulated SGs (**Figure 6**). Consistent with this notion are previous observations that documented LC3 location and release by bone marrow derived MCs (38). We do not presently know if cytosolic LC3 binds to the macropinosomes, in analogy to LC3-associated macropinocytosis during membrane repair (37) or LC3-associated phagocytosis (45), in which case, the LC3-GFP-positive, but NPY-negative vesicles correspond to macropinosome derived vesicles, that fuse with the type I SGs to form type IIa SGs (**Figure 6**), or whether LC3 resides on phagophores or autophagosomes, that respectively engulf or fuse with macropinosome-derived vesicles, prior to their fusion with the SGs to form type IIb SGs (**Figure 6**), in analogy to the biogenesis of lamellar bodies in the lung (46). Future studies will distinguish between these possibilities. It is interesting to note in this context that autophagy has been implicated in playing a role in FcεRI-mediated exocytosis (38) and we have shown that 3-methyladenine (3-MA), an inhibitor of canonical autophagy, inhibits MRGPRX2-mediated secretion (21). However, while these results implicate canonical autophagy in the mechanism of MC exocytosis, they do not necessarily counteract the possibility of non-canonical autophagy playing a role in MRGRPX2 trafficking.

In summary, deciphering the spatiotemporal regulation of MRGPRX2 is of particular interest given the different ability of MRGPRX2 ligands to stimulate internalization. Thus, it is tempting to speculate that biased ligands, such as AG30/5C (11), that do not stimulate receptor internalization, and therefore restrict MRGPRX2 signaling to the plasma membrane, may confer distinct functions than balanced ligands, such as SP (25), that may stimulate intracellular signaling. Delineating the route of post endocytic trafficking of MRGPRX2 may thus pave the road for the rational design of cell-permeable antagonists that will specifically target undesired and site-specific MRGPRX2 activation. Here we show that SP-bound MRGPRX2 internalizes by two parallel pathways, including endocytosis and macropinocytosis. We also show that the internalized receptor is targeted to internal non-degradative compartments that include the SGs. Further, we uncovered a novel mechanism that connects macropinosomes and SGs and which mediates the delivery of MRGPRX2 to the SGs. Whether the SGs participate in MRGPRX2 signaling or serve as an alternative to endocytic recycling, for the direct coupling of receptor retrieval with exocytosis, are intriguing questions, which we shall address in our future studies.

## Data Availability Statement

The original contributions presented in the study are included in the article/**Supplementary Material**. Further inquiries can be directed to the corresponding author.

## Author Contributions

RS-E and HA conceived the project, PL-H and EK designed and performed the experiments, analysed the data, discussed the results, PL-H prepared the figures and commented on the manuscript, RS-E supervised the project and wrote the manuscript. All authors reviewed the manuscript. All authors contributed to the article and approved the submitted version.

## Funding

This work was supported by grant 1600/19 from the Israel Science Foundation, founded by the Israel Academy for Sciences (to RS-E), and grant 2017182 by the United States– Israel Binational Science Foundation (to RS-E). PL-H was supported through The American Association of Immunologists Careers in Immunology Fellowship Program. This work was also supported by National Institutes of Health grant R01-AI124182 (to HA).

## Conflict of Interest

The authors declare that the research was conducted in the absence of any commercial or financial relationships that could be construed as a potential conflict of interest.

## Publisher’s Note

All claims expressed in this article are solely those of the authors and do not necessarily represent those of their affiliated organizations, or those of the publisher, the editors and the reviewers. Any product that may be evaluated in this article, or claim that may be made by its manufacturer, is not guaranteed or endorsed by the publisher.

## Supplementary Material

Supplementary Video 1 RBL-MRGPRX2 cells were transiently transfected with NPY-mRFP, marking SGs, and LC3-GFP. Next day, cells were triggered for 15 min with 10µM SP and imaged using a Zeiss LSM 710 confocal microscope. Three-dimensional reconstructions of z stacks (slice thickness ≤ 0.5µm for both fluorescent channels with ~ 10 slices) were performed by using Imaris software (Bitplane, Zurich, Switzerland).

Supplementary Figure 1 RBL-MRGPRX2 cells **(A, B)** or LAD-2 cells **(C, D)** were stained with anti-MRGPRX2 antibody followed by Alexa Fluor® 647 secondary antibody (blue lines) or by the secondary antibody (grey lines, control) in the absence **(A, C)** or presence **(B, D)** of 0.1% saponin, and analyzed by flow cytometry.

Supplementary Figure 2 LAD-2 cells were either left untreated (UT) or triggered with 10 µM SP for 30 min (n = 2 with at least 20 cells/condition per experiment). Cells were immunostained with mouse anti-MRGPRX2 and rabbit anti-syntaxin 3 (STX3) antibodies, followed by Alexa Fluor® 647-conjugated goat anti-mouse secondary antibodies (pseudo colored cyan) and Alexa Fluor® 488-conjugated goat anti-rabbit secondary antibodies (pseudo colored red). Enlargements correspond to the boxed areas. Enlargements correspond to the boxed areas. Macropinosomes are shown by the overlap with the brightfield (BF) image. White arrows point to MRGPRX2 that is localized to macropinosomes. Blue arrows points to STX3 located at the plasma membrane, where it overlaps with MRGPRX2. Arrowheads point to macropinosomes. Scale bars = 10µm.

## Abbreviations

MC, Mast cell; SP, substance P; SG, secretory granule; TGN, trans Golgi network; IgE, immunoglobulin; GPCRs, G protein coupled receptors; Mrgprs, Mas-related G protein coupled receptors; CytD, cytochalasin D; FACS, flow cytometric analyses; MFI, mean fluorescence intensity; NK1, Neurokinin 1; DN, dominant negative; 3-MA, 3-methyladenine: UT, untreated; BF, brightfield; HA, hemagglutinin.
