# Supplementary figures and images for: Spatiotemporal Patterns of Substance P-Bound MRGPRX2 Reveal a Novel Connection Between Macropinosome Resolution and Secretory Granule Regeneration in Mast Cells
Source: Front Immunol. 2022 Jun 28;13:892239. doi: 10.3389/fimmu.2022.892239 (PMC9273857; doi:10.3389/fimmu.2022.892239)

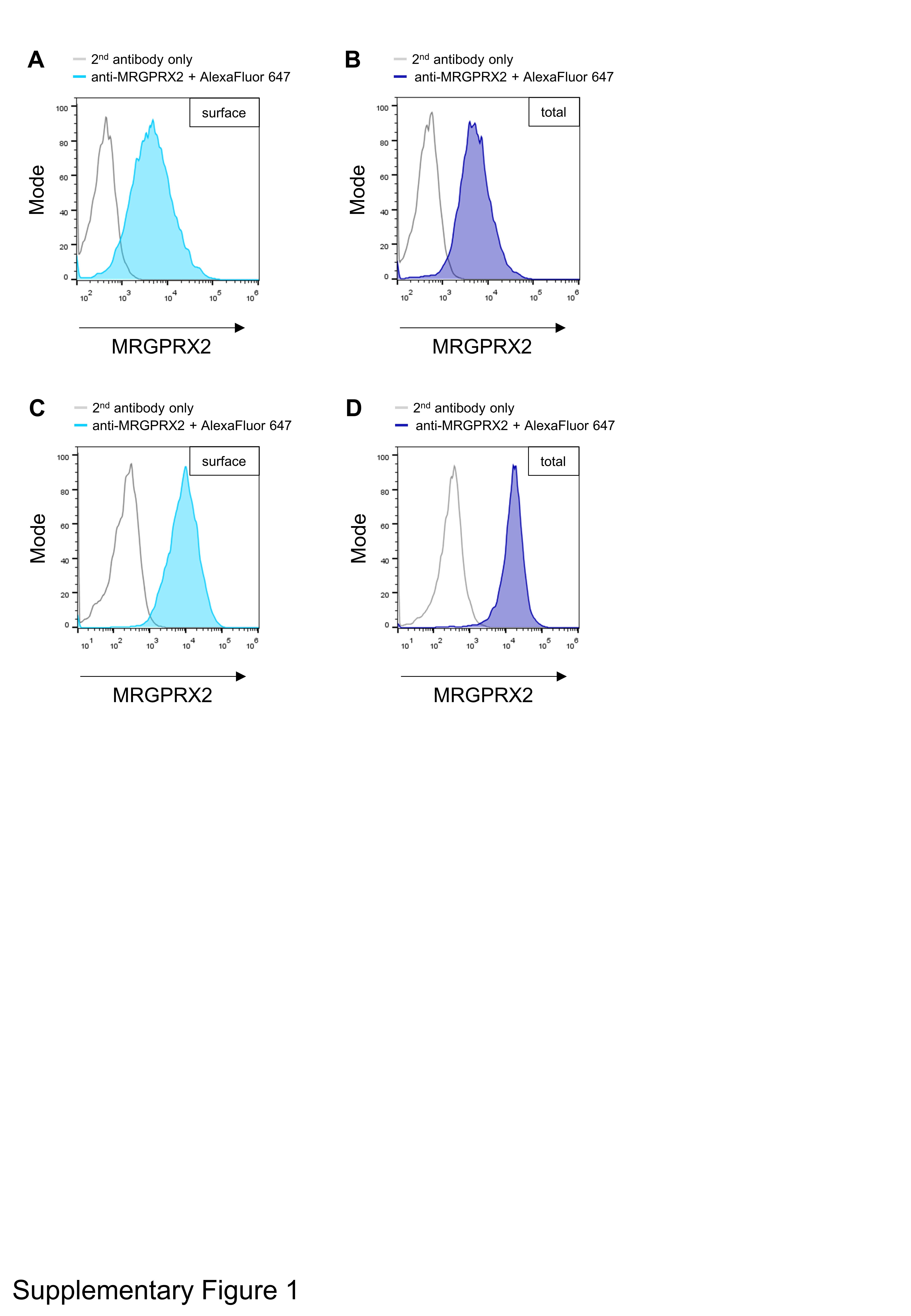

Supplement: Supplementary Figure 1 — RBL-MRGPRX2 cells (A, B) or LAD-2 cells (C, D) were stained with anti-MRGPRX2 antibody followed by Alexa Fluor® 647 secondary antibody (blue lines) or by the secondary antibody (grey lines, control) in the absence (A, C) or presence (B, D) of 0.1% saponin, and analyzed by flow cytometry. [file Image_1.tif]

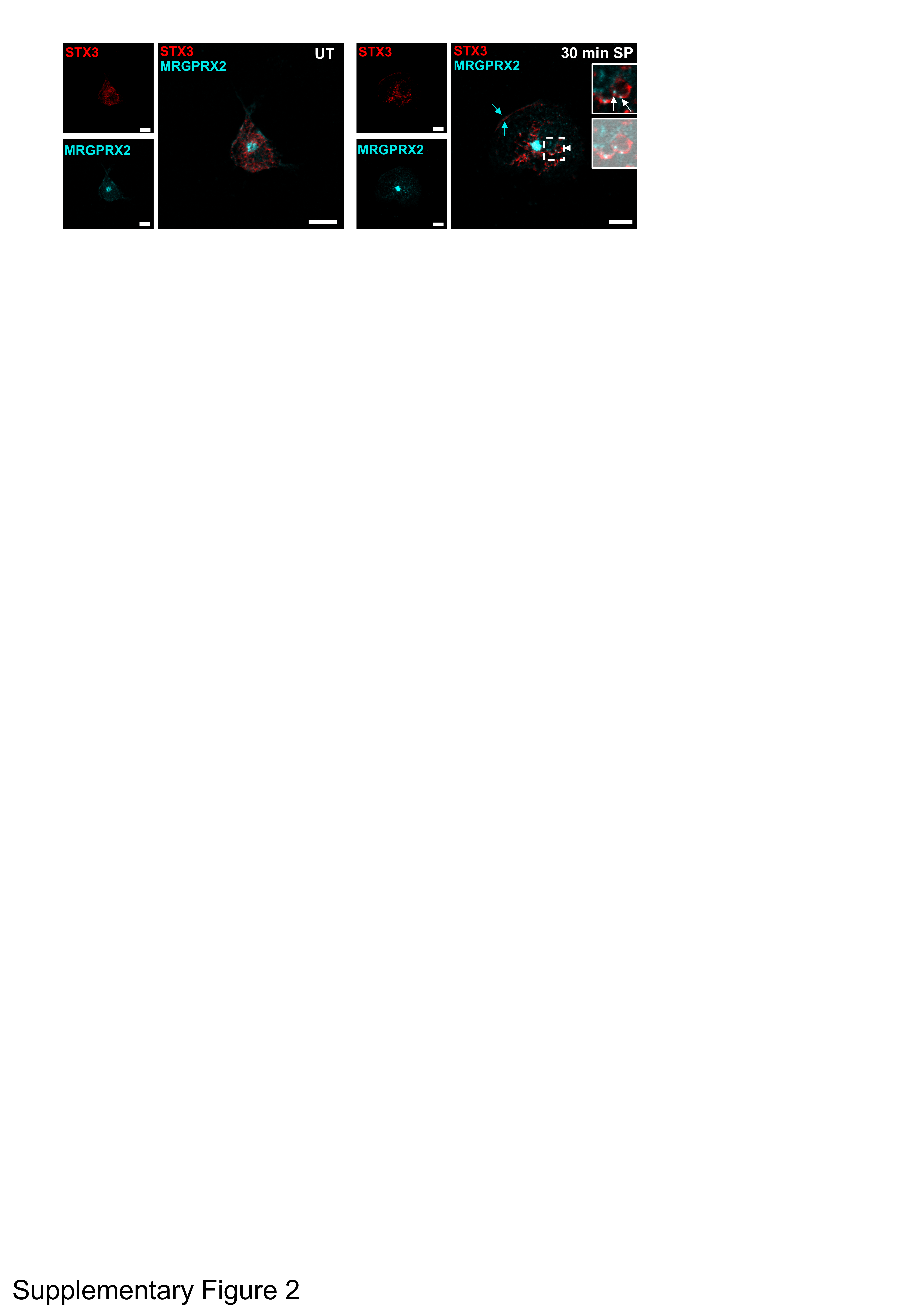

Supplement: Supplementary Figure 2 — LAD-2 cells were either left untreated (UT) or triggered with 10 µM SP for 30 min (n = 2 with at least 20 cells/condition per experiment). Cells were immunostained with mouse anti-MRGPRX2 and rabbit anti-syntaxin 3 (STX3) antibodies, followed by Alexa Fluor® 647-conjugated goat anti-mouse secondary antibodies (pseudo colored cyan) and Alexa Fluor® 488-conjugated goat anti-rabbit secondary antibodies (pseudo colored red). Enlargements correspond to the boxed areas. Enlargements correspond to the boxed areas. Macropinosomes are shown by the overlap with the brightfield (BF) image. White arrows point to MRGPRX2 that is localized to macropinosomes. Blue arrows points to STX3 located at the plasma membrane, where it overlaps with MRGPRX2. Arrowheads point to macropinosomes. Scale bars = 10µm. [file Image_2.tif]
